# A Study on Machine Learning-Based Feature Classification for the Early Diagnosis of Blade Rubbing

**DOI:** 10.3390/s24186013

**Published:** 2024-09-17

**Authors:** Dong-hee Park, Byeong-keun Choi

**Affiliations:** 1 DAVISS Inc., Jinju-si 52828, Republic of Korea; daviss.dhp@gmail.com; 2 Department of Energy and Mechanical Engineering, Gyeongsang National University, Tongyeong-si 53064, Republic of Korea

**Keywords:** machine learning, feature-based diagnosis, blade rubbing, turbine blade, signal preprocessing, bandpass filter, band reject filter, diagnosis

## Abstract

This research focuses on the development of a machine learning-based approach for the early diagnosis of blade rubbing in rotary machinery. In this paper, machine learning-based diagnostic methods are used for blade rubbing early diagnosis, and the faults are simulated using experimental models. The experimental conditions were simulated as follows: Excessive rotor vibration is generated by an unbalance mass, and blade rubbing occurs through excessive rotor vibration. Additionally, the severity of blade rubbing was also simulated while increasing the unbalance mass. And then, machine learning-based diagnostic methods were applied and the trends according to the severity of blade rubbing were compared. This paper provides a signal processing method through feature analysis to diagnose blade rubbing conditions in machine learning. It was confirmed that the results of the unbalance and blade rubbing represent different trends, and it is possible to distinguish unbalance from blade rubbing before blade rubbing occurs. The diagnosis using machine learning methods will be applicable to rotating machinery faults like blade rubbing; furthermore, the early diagnosis of blade rubbing will be possible.

## 1. Introduction

Gas turbines are widely used in various industrial fields such as aircraft, ships, and nuclear and thermal power generation. Gas turbines used in the power generation industry are composed of motors, compressors, combustors, turbine blades, and generators. Turbine blades function within the most extreme conditions of heat and high pressure among these components, playing a pivotal role in the conversion of fluid energy into mechanical energy [1]. Hence, in the event of a fault, it becomes imperative to promptly identify its root cause to facilitate immediate remedial action.

In power generation facilities, gas turbines are generally used to reduce the gap between blades and casing to improve energy efficiency [2]. However, if the gap between the blade and the casing is reduced, there is a very high possibility of a rubbing phenomenon occurring in contact with the casing, which is the fixed part, during operation. This phenomenon can lead to the damage and abrasion of the casing and blades, which can reduce the efficiency of the turbine. Furthermore, within the spectrum of faults found in gas turbines used for power generation, blade-related issues constitute the highest proportion at 42% [3], with blade rubbing being a notable concern. This blade rubbing is substantially caused by excessive shaft vibration, distortion due to thermal stress, and misalignment, and on the contrary, the rubbing itself may cause other types of faults [4,5,6,7]. In severe cases, the blade may fall off and affect other facilities, thereby increasing repair costs and the risk of accidents.

Vibration monitoring is performed to diagnose faults in facilities including blade rubbing, which is the easiest and most used method for diagnosis because it can check the structure, operation condition, and dynamic behavior of the facility [8,9]. The characteristics of the rubbing vibration signal show a truncated shape in the time waveform, and, in the FFT Spectrum, a fractional harmonic composition of the driving (rotation) component is generated [10]. However, the friction force generated by blade rubbing is less than the mass of the shaft. As this friction force is transmitted to the shaft, the energy is attenuated. Therefore, unlike shaft rubbing, it is difficult to estimate fault characteristics through signals intuitively [11,12].

Turbines generally measure the displacement of the shaft. Therefore, shaft rubbing is easy to detect because it shows the characteristics well. However, blade rubbing may not be detected in the signal. Therefore, research and development on the early diagnosis of blade rubbing is needed to solve the problems of the stability, cost, and efficiency of turbines [13,14].

This study was evaluated by applying a machine learning-based diagnostic method to perform blade rubbing of early diagnosis that may occur in turbines. The study model utilized a self-manufactured rubbing test device (RTD). Using this, experiments were conducted to simulate blade friction phenomena occurring in real-field conditions by gradually increasing the unbalance mass. A total of 18 experimental cases were conducted, comprising unbalance and rubbing tests. The unbalance mass varied depending on the case, with equal increments applied to both tests. Cases 1 to 4 had an unbalance mass ranging from 0 to 1.5 g with a difference of 0.5 g. For Cases 5 to 9, the mass was increased by 0.1 g for each, ranging from 1.6 to 2.0 g. In the case of rubbing, rubbing occurred from 1.6 g, and rubbing was confirmed using a thermal imaging camera. This data set was applied to machine learning diagnosis with the intention to confirm the possibility of blade rubbing early diagnosis through performing tendency evaluation and classification according to deterioration (increased balance mass and increased rubbing severity).

## 2. Experiment

### 2.1. Experimental Model and Data Acquisition

**Figure 1:**
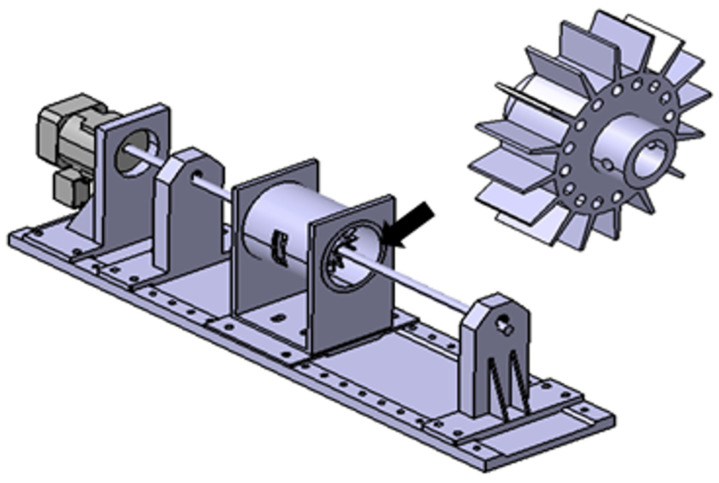
Test-rig (rubbing test device, RTD).

**Figure 2:**
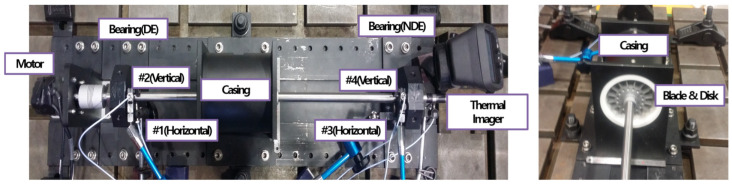
Experimental model.

The research model used a self-made rubbing test device to simulate blade rubbing based on excessive axial vibration and is composed of a hole, blade, casing, shaft, roller bearing and motor. The experiment was conducted to gradually increase the unbalanced mass in the hole of each disk to generate blade rubbing. The number of holes and blades was selected as 16, and the driving speed was about 3500 rpm. See the research model in Figure 1 and Figure 2.

**Figure 3:**
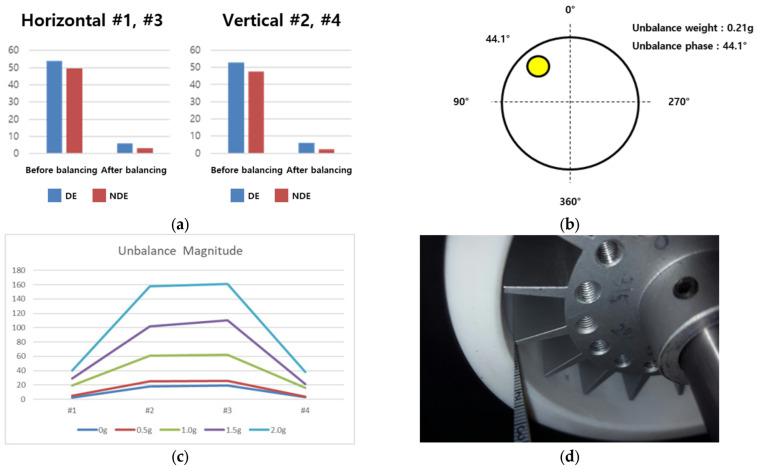
Experimental setting process: (**a**) comparison of amplitude; (**b**) residual unbalance magnitude and phase; (**c**) unbalance response according to mass; (**d**) clearance adjustment of blade and casing.

First, balancing is performed in consideration of the influence factor so that faults other than those to be simulated do not occur as much as possible in advance. After that, to check whether balancing is performed, the size of the residual inequality is checked, and the phase of the residual inequality is checked so that the appropriate response comes out as the degree of inequality increases. And then, the response is checked according to the degree of inequality and the gap is adjusted between the blade and casing based on the response size. The setting process as above is shown in Figure 3.

**Figure 4:**
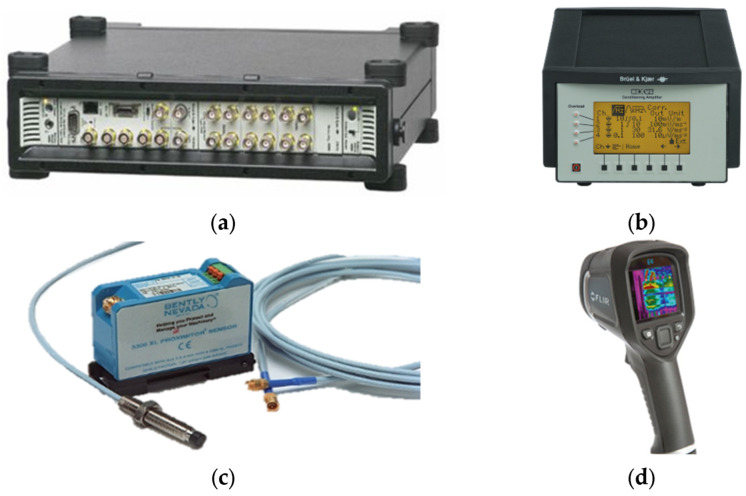
Data acquisition equipment: (**a**) Pulse 3560C; (**b**) Nexus amplifier; (**c**) displacement sensor; (**d**) thermal imager.

To obtain the data in the normal, single, and complex fault states, the data used a vibration precision analysis device, Pulse 3560C (B&K), a displacement sensor, and a thermal imaging camera. In this case, a thermal imaging camera was used to check the degree of contact with blade friction. The sampling rate of the acquisition device is 65,536 Hz, and the data acquisition time for each state is 30 s. Figure 4 and Table 1 show the measurement equipment photographs and the specifications of the measurement equipment.

### 2.2. Experimental Method and Case

**Figure 5:**
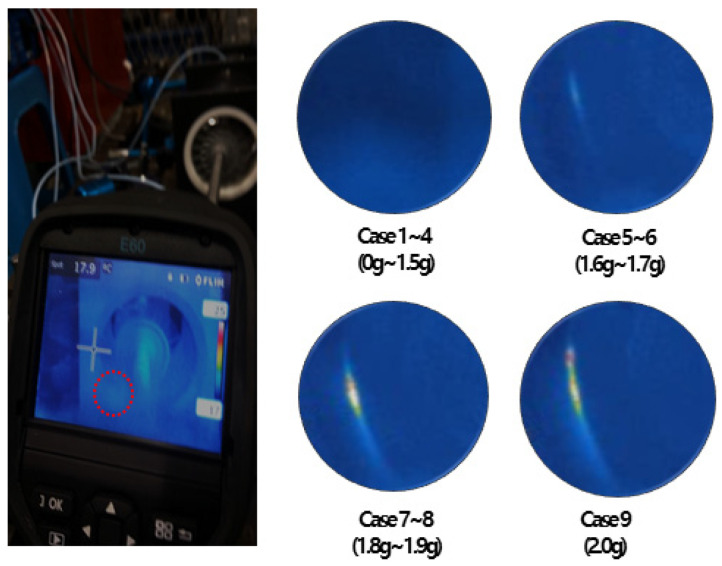
Blade rubbing contact state.

**Figure 6:**
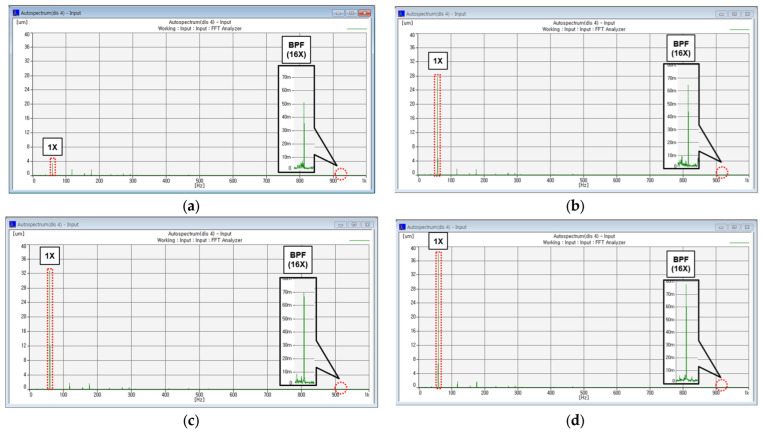
FFT Spectrum: (**a**) Case 1 (Unbalance mass: 0 g) #4; (**b**) Case 5 (Unbalance mass: 1.6 g) #4; (**c**) Case 7 (Unbalance mass: 1.8 g) #4; (**d**) Case 9 (Unbalance mass: 2.0 g) #4.

There are a total of 18 normal and fault data simulated, and the entire case is shown in Table 2 below. The simulated fault obtained data through gradually increasing the degree of unbalance to simulate blade rubbing caused by excessive axial vibration, and the degree of blade rubbing contact was confirmed through a thermal imaging camera as shown in Figure 5. Cases 1–4 are not in contact, Cases 5–6 are in fine contact (contact with 1.6 g to 1.7 g of inequality), Cases 7–8 are in contact (contact with 1.8 g to 1.9 g of inequality), and Case 9 is in excessive contact (contact with 2.0 g of inequality). The FFT Spectrum was additionally checked to confirm whether the fault data were properly simulated. Figure 6 shows the FFT Spectrum of the simulated fault data in non-contact, fine contact, contact, and excessive contact state.

As a result, as the unbalance mass increases, for an amplitude of about 58 Hz, the 1X component grows in the FFT Spectrum, and the amplitude of the 928 Hz (58 Hz × 16) component related to blade rubbing grows according to the size of the unbalanced mass (500 nm -> 780 nm). However, it cannot be diagnosed as a blade fault because it has a microscopic amplitude of less than 1 um. In addition, it is confirmed that the fractional multiple harmonic component, which is the vibration characteristic of the rubbing fault, also does not appear. This is because the manufactured rubbing test device casing inside is made of PTFE (Poly-tetrafluoroethylene) material, which is less rigid than the blade’s (stainless), and it is considered that the characteristics of excessive contact such as reverse precession do not occur. The PTFE’s lower rigidity and friction reduce the intensity of contact events, preventing excessive vibrational phenomena. As a result, the typical fractional frequencies associated with rubbing faults are not observed.

As such, it must be determined whether the possibility of state classification according to each fault type and the degree of deterioration of the fault can be expressed; the tendency of fault deterioration can be expressed by applying the machine learning diagnostic method to a fault condition in which actual rubbing occurs (check the thermal imaging camera), but the fault characteristics do not occur in the FFT Spectrum.

## 3. Proposed Diagnosis Methodology

**Figure 7:**
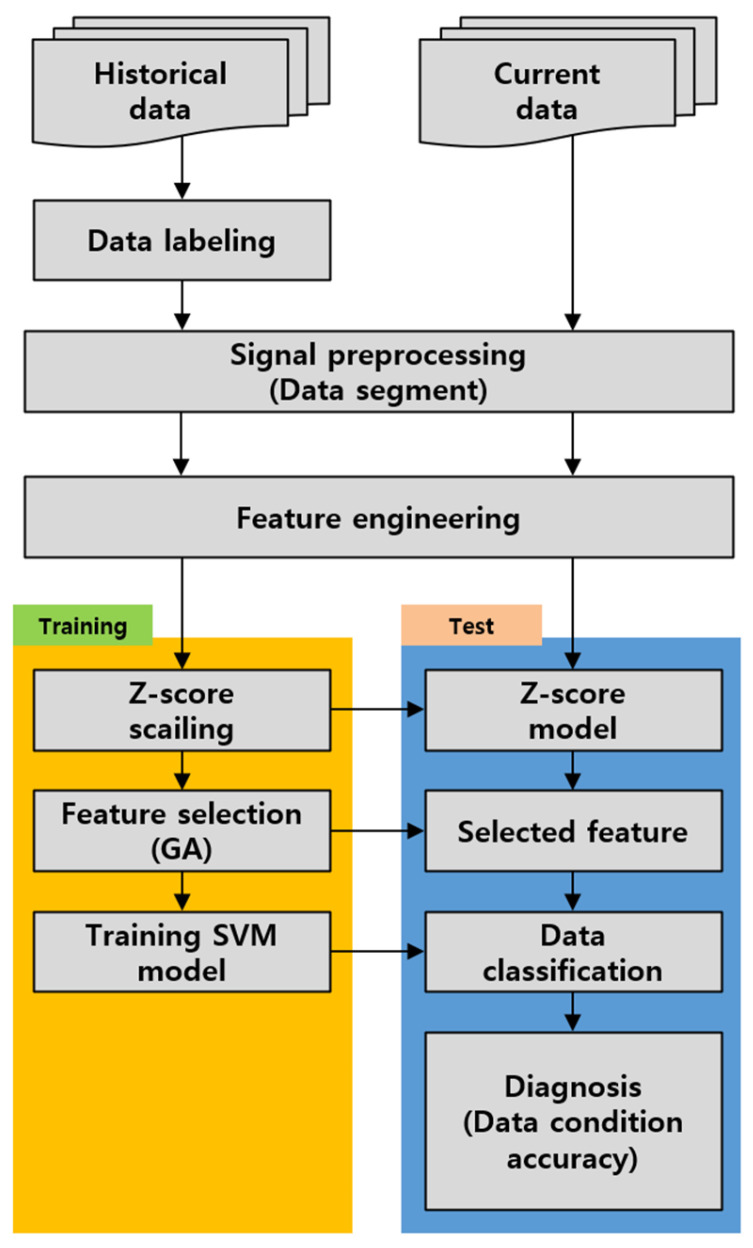
Machine learning diagnosis process.

Supervised learning based on shape features and statistical machine learning is carried out following the procedure shown in Figure 7 [11,12]. First, after labeling the historical data, the data obtained from the rotating machine, being periodic signals, are segmented into 1 s intervals to secure a sufficient amount of training data and are used for evaluation [15,16,17]. And then, as shown in Table 3, the sampled data calculate a total of 30 features, including time-domain features (19), entropy-domain features (4), and frequency-domain features (7), which encompass both the statistical and shape information of the signal.

In the training step, since the calculated features may have different scale sizes, bias with feature sizes may occur when selecting features. Therefore, the normalization used a z-score extending to a Gaussian distribution with a mean of zero and a standard deviation of one. Furthermore, when too many features are used for training, unclassifiable data may also be included in the training process. To resolve the Genetic Algorithm (GA) [16,17,18,19], an optimization algorithm that mimics natural evolution is applied. The objective function selects the optimal combination of three features where the intra-class clustering is high, and the distance between different states is maximized. The selected three feature combinations are then used to form a feature space and create decision boundaries through SVMs [20,21,22,23]. In the test step, for diagnosing current data, the same preprocessing steps are applied to calculate the features. Z-score normalization and the selected features used during training are applied to the current data, which is then input into the trained SVM model to derive the diagnosis results.

## 4. Result

### 4.1. Machine Learning Classification Analysis

**Figure 8:**
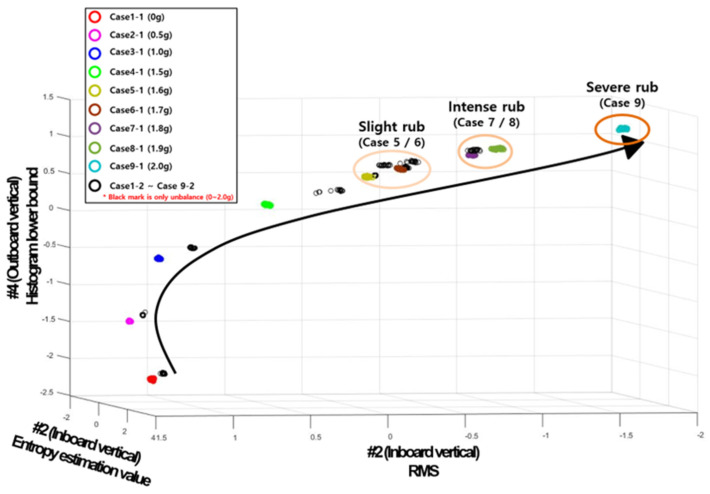
Application results of machine learning diagnosis.

Figure 8 shows the result of diagnosing the condition by applying the experimental data to machine learning. In the upper left of Figure 8, the simulated normal and defective cases are displayed as legends. The results expressed in the primary colors of Cases 1-1–9-1 in the upper right indicate the feature of the blade rubbing experimental data by unbalance, and in the case of the black color (Cases 1-2~9-2), this indicates the feature of the unbalanced experimental data. In this paper, the data in which only the unbalance is simulated refers to data acquired after removing the rubbing test device casing.

As a result of the machine learning application, it can be seen that in both experimental data cases, as the unbalanced mass increases, the sampled feature values tend to grow (decrease or increase) gradually, as indicated by arrows. However, it was confirmed that rubbing and unbalance fault data samples appeared similarly. In this case, if the data sample for each case increases, the performance of distinguishing between unbalance and rubbing data samples will decrease. The reason for this is confirmed to be that the 1X component is the most dominant in the raw signal and no other fault features appear when analyzing the data of all experimental data. Therefore, similar features were calculated in the feature engineering (calculation) process. Therefore, a new method was applied for the clear classification and trend expression of rubbing data samples and unbalance data samples.

### 4.2. Machine Learning Trend Analysis

**Figure 9:**
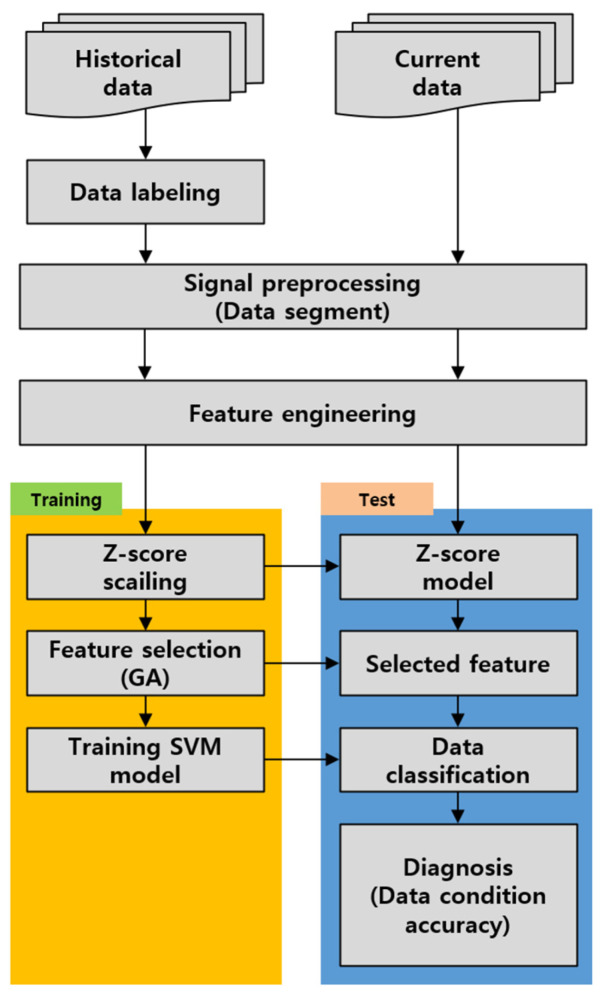
The newly conducted machine learning diagnosis process.

**Figure 10:**
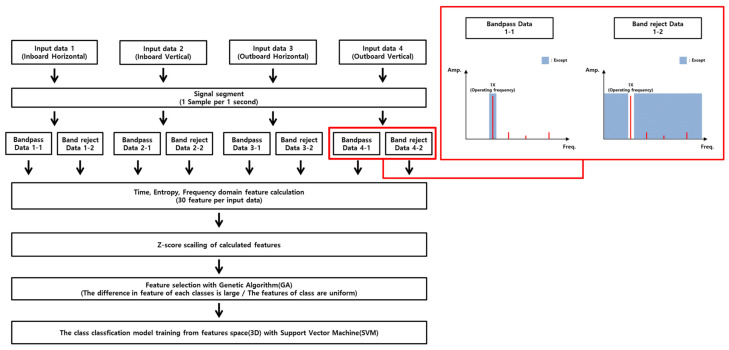
Signal segmentation detailed conceptual diagram.

Figure 9 shows the process of machine learning diagnostics that added a signal preprocessing process to improve the limitations identified in the previous machine learning classification analysis. Figure 10 is a flowchart to explain the added signal processing. Data including only 1X component (1X bandpass signal), which is an unbalanced fault characteristic identified during data analysis, and the data of signals (1X band reject signal) excluding the unbalanced fault characteristic were extracted. Therefore, the existing four input data were reorganized into eight input data through signal processing and were applied to machine learning. For the reconstructed input data, a total of 240 features (8 input X 30 features) were calculated through the feature engineering process. Because this method calculates features from reconstructed input data (the most dominant 1X components and other components), features that can clearly distinguish between unbalance and rubbing experimental cases are likely to be selected.

**Figure 11:**
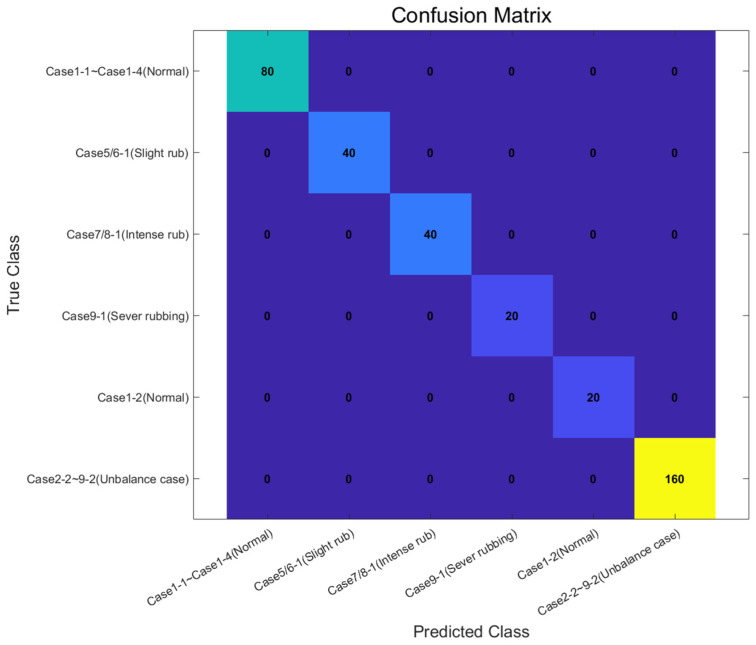
Confusion matrix results of machine learning diagnosis (new method).

Figure 11 shows the results of the new machine learning method represented in a confusion matrix. As mentioned in Table 2, under the “Cover on” condition, Cases 1-1 to 4-1 were designated as normal, Cases 5-1 to 6-1 as slight rubbing, Cases 7-1 to 8-1 as intense rubbing, and Cases 9-1 as severe rubbing. Under the “Cover off” condition, Cases 1-2 to 3-2 were learned as normal and Cases 4-2 to 9-2 as unbalance, resulting in a total of six classes for training. Although the number of training data for each class differs, the classification results show 100% accuracy.

**Figure 12:**
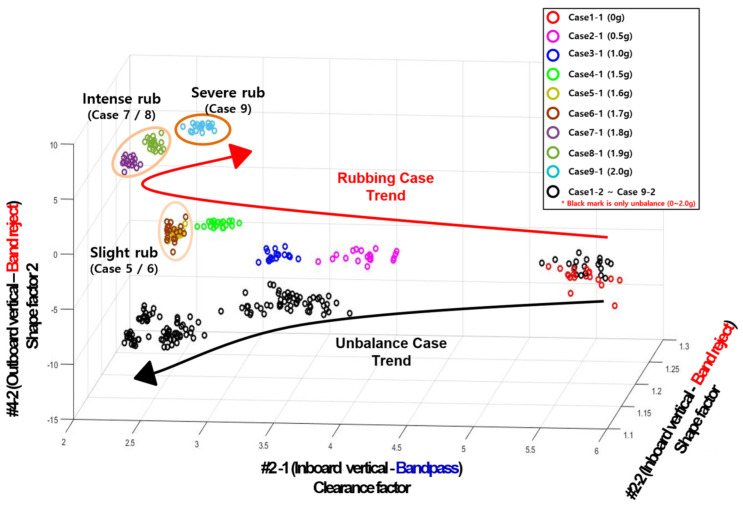
Application results of machine learning diagnosis (new method).

To explain more intuitively, Figure 12 is the result of the new method of machine learning, which visually represents the classification. Unlike before, the unbalance and rubbing experimental classes are completely classified. Among the three features selected through GA, two features extracted from the band reject signal (#4-2 shape factor 2 and #2-2 shape factor) are selected. This is like saying that there are statistical features that can distinguish between rubbing and the unbalance in areas other than 1X components. In addition, it can be seen that rubbing and unbalance experimental data samples are expressed with arrow-like tendencies, respectively, as the unbalanced mass increases in the configured 3D feature area.

To analyze the results, it can be confirmed that Case 1-1 (rubbing experimental 0 g) and Case 1-2 (unbalance experimental 0 g) are clustered equally, regardless of the three selected characteristics. Cases 2-1 to 9-1 are clustered the same as unbalance for the X-axis (#2-1 clearance factor, 1X bandpass filter) characteristics. (If only the X direction is considered, the X-axis feature values decrease in the unbalanced mass increase, but clusters in the same area by considering only the mass used for each case). Cases 2-1 to 9-1 are clustered the same as unbalance for the X-axis (#2-1 clearance factor, 1X bandpass filter) characteristics. (If only the X direction is considered, the X-axis characteristic decreases in the unbalance mass, but clusters in the same area by considering only the mass used for each case). However, it can be confirmed that the Y-axis (#2-2 shape factor, 1X band reject filter) and Z-axis (#4-2 shape factor 2, 1X band reject filter) features are clearly distinguished between rubbing and unbalance (the degradation trend in the feature manifests in a completely opposite direction).

## 5. Conclusions

The purpose of this study was to confirm the possibility of an early diagnosis of blade rubbing occurring in a rotating body by applying machine learning diagnostic technology. The research model used a self-made rubbing test device. The equipment was manufactured in a form capable of adjusting and detaching the casing clearance, and the shape in which excessive shaft vibration develops into blade rubbing due to an increase in unbalance was simulated. The experiment was carried out in two ways. The first was to gradually increase the unbalanced mass after removing the casing. This is an unbalanced deterioration experiment. Second, after attaching the casing, the form in which the blade and casing rubbed according to the increase in the unbalanced mass was simulated. The conditions for increasing the unbalanced mass are the same as those of the first experiment. Rubbing began from the unbalance mass of 1.6 g and was the most severe at 2.0 g. Whether or not rubbing occurred was confirmed through a thermal imaging camera and an FFT Spectrum. The reason for conducting the two experiments was to check whether it is possible to perform fault condition classification and to express the fault deterioration (trend) when applied to machine learning diagnosis.

As a result of applying the existing machine learning diagnostic technology, it was confirmed that the unbalance data and blade rubbing data tended to gradually increase the calculated features according to the deterioration of the fault. However, it shows a similar tendency to the results of the data simulating only unbalance, and it is not easy to distinguish whether it is increased by blade rubbing or unbalance. Accordingly, signal processing was performed on the input data to classify the fault characteristics in the signal and was then applied to machine learning diagnosis. As a result, it was confirmed that blade rubbing and unbalance conditions were classified and that even the deterioration of faults could be represented.

The overall research content of this paper is summarized as follows:

- Using the research model, the rubbing test device, a total of 30 features in the area of time, frequency, and entropy were used for machine learning-based diagnosis. As a result of machine learning diagnosis, it was confirmed that the blade rubbing severity could be evaluated, but it was similar to the unbalance experiment data. This is because the 1X component is the most dominant in all signals measured through the experiment and the size of other fault characteristics (BPF) is small. This is because the values of statistical features calculated in the feature engineering process do not differ or are small when the entire original signal is considered. Therefore, before applying to machine learning, signal processing was performed to divide input data into two types: 1X component data including unbalanced information and data excluding (but including a range of other fault characteristics) 1X component. As a result, it can be confirmed that the case of simulating only unbalance and the case of blade rubbing caused by unbalance have different tendencies and are classified before blade rubbing occurs.
- Machine learning diagnostic methods derive different results depending on input data or calculation and selected features. To increase the performance of machine learning diagnostic technology, it is essential to develop appropriate features that can classify each fault or apply a data preprocessing method that can extract the features of the fault condition well.
- We will use other fault data sets such as misalignment or bearing fault later. We will develop a general purpose new machine learning diagnostic process that extracts and applies the characteristics of each fault condition.

## Figures and Tables

**Table 1:** Specification of sensor and acquisition condition.

Equipment	Detail
Pulse 3560C (Brüel & Kjær, Nærum, Denmark)	4/2-ch Input/output Module Operating Freq. range: 0~25.6 kHz
Displacement sensor 3300 (Bently Nevada, Minden, NV, USA)	Operating Freq. range: 1~10 kHz Sensitivity: 7.87 V/mm
Amplifier 2690 (Brüel & Kjær, Nærum, Denmark)	Displacement (optional): 1.0 Hz to 1 kHz INHERENT NOISE (2 Hz TO 22.4 kHz)
Thermal imager camera (Flir, Wilsonville, OR, USA)	IR Resolution: 140 × 120 Thermal sensitivity: <0.06 °C

**Table 2:** Experimental case.

Rubbing Experiment (Cover On)			Unbalance Experiment (Cover Off)		
Case	Unbalance Mass	Condition	Case	Unbalance Mass	Condition
Case 1-1	0 g	Normal	Case 1-2	0 g	Normal
Case 2-1	0.5 g		Case 2-2	0.5 g	
Case 3-1	1.0 g		Case 3-2	1.0 g	
Case 4-1	1.5 g		Case 4-2	1.5 g	Unbalance
Case 5-1	1.6 g	Slight rubbing	Case 5-2	1.6 g	
Case 6-1	1.7 g		Case 6-2	1.7 g	
Case 7-1	1.8 g	Intense rubbing	Case 7-2	1.8 g	
Case 8-1	1.9 g		Case 8-2	1.9 g	
Case 9-1	2.0	Severe rubbing	Case 9-2	2.0	

**Table 3:** Statistical feature formula.

Features	Definition (Equation)	Features	Definition (Equation)
Peak	$f_{1}=\max(\left|x\right|)$	Kurtosis factor	$f_{16}=\frac{\frac{1}{N}\sum_{n=1}^{N}{\left(\frac{x_{n}-\bar{x}}{\sigma }\right)}^{4}}{{\left(\frac{1}{N}\sum_{n=1}^{N}{x_{n}}^{2}\right)}^{2}}$
Peak to Peak	$f_{2}=\max\left(x\right)-\min(x)$	Smoothness	$f_{17}=1-\left(1+{\left(\sqrt{\frac{1}{N}\sum_{n=1}^{N}(x_{n}-{x)}^{2}}\right)}^{2}\right)$
Mean	$f_{3}=\frac{1}{N}\sum_{n=1}^{N}\left|x_{n}\right|$	Uniformity	$f_{18}=1-\left(\frac{\sqrt{\frac{1}{N}\sum_{n=1}^{N}{\left(x_{n}-x\right)}^{2}}}{\frac{1}{N}\sum_{n=1}^{N}x_{n}}\right)$
Standard deviation	$f_{4}=\sqrt{\frac{1}{N}\sum_{n=1}^{N}(x_{n}-{x)}^{2}}=\left|\sigma \right|$	Normal negative log-likelihood	$f_{19}={\frac{1}{\sigma \sqrt{2\pi }}e}^{\frac{-{\left(x-\mu \right)}^{2}}{2\sigma^{2}}}$
Root Mean Square	$f_{5}=\sqrt{\frac{1}{N}\sum_{n=1}^{N}{x_{n}}^{2}}$	Entropy estimation value	$f_{20}=-\sum_{n=1}^{N}P_{n}\cdot \ln P_{n}$
Kurtosis	$f_{6}=\frac{1}{N}\sum_{n=1}^{N}{\left(\frac{x_{n}-\bar{x}}{\sigma }\right)}^{4}$	Frequency center	$f_{21}=\frac{1}{N}\sum_{n=1}^{N}\left|s_{n}\right|$
Crest factor	$f_{7}=\frac{\max(\left|x_{n}\right|)}{\sqrt{\frac{1}{N}\sum_{n=1}^{N}{x_{n}}^{2}}}$	Mean square frequency	$f_{22}=\frac{\int_{0}^{\infty }f^{2}s\left(f\right)df}{\int_{0}^{\infty }s\left(f\right)df}$
Clearance factor	$f_{8}={\left(\frac{\max(\left|x\right|)}{\frac{1}{N}\sum_{n=1}^{N}\sqrt{\left|x_{n}\right|}}\right)}^{2}$	RMS frequency	$f_{23}=\sqrt{\frac{\int_{0}^{\infty }f^{2}s\left(f\right)df}{\int_{0}^{\infty }s\left(f\right)df}}$
Impulse factor	$f_{9}=\frac{\max(\left|x\right|)}{\frac{1}{N}\sum_{n=1}^{N}\sqrt{\left|x_{n}\right|}}$	Variance frequency	$f_{24}=\frac{\int_{0}^{\infty }{\left(f-\frac{\int_{0}^{\infty }fs\left(f\right)df}{\int_{0}^{\infty }s\left(f\right)df}\right)}^{2}s\left(f\right)df}{\int_{0}^{\infty }s\left(f\right)df}$
Shape factor	$f_{10}=\frac{\sqrt{\frac{1}{N}\sum_{n=1}^{N}{x_{n}}^{2}}}{\frac{1}{N}\sum_{n=1}^{N}\left|x_{n}\right|}$	Root variance frequency	$f_{25}=\sqrt{\frac{\int_{0}^{\infty }{\left(f-\frac{\int_{0}^{\infty }fs\left(f\right)df}{\int_{0}^{\infty }s\left(f\right)df}\right)}^{2}s\left(f\right)df}{\int_{0}^{\infty }s\left(f\right)df}}$
Skewness	$f_{11}=\frac{1}{N}\sum_{n=1}^{N}{\left(\frac{x_{n}-\mu }{\sigma }\right)}^{3}$	Spectrum overall	$f_{26}=\sum_{n=1}^{N}S_{n}$
Square Mean Root	$f_{12}={\left(\frac{1}{N}\sum_{n=1}^{N}\sqrt{\left|x_{n}\right|}\right)}^{2}$	Spectrum RMS overall	$f_{27}=\sqrt{\sum_{n=1}^{N}{S_{n}}^{2}}$
5th Normalized Moment	$f_{13}=\frac{1}{N}\sum_{n=1}^{N}{\left(\frac{x_{n}-\bar{x}}{\sigma }\right)}^{5}$	Entropy estimation error value	$f_{28}=-\sum_{n=1}^{N}P_{n}\cdot \ln{P_{n}}^{2}$
6th Normalized Moment	$f_{14}=\frac{1}{N}\sum_{n=1}^{N}{\left(\frac{x_{n}-\bar{x}}{\sigma }\right)}^{6}$	Histogram Upper bound	$f_{29}=\max\left(x_{i}\right)+\frac{\left(\max\left(x_{i}\right)-min\left(x_{i}\right)\right)}{2(n-1)}$
Shape factor 2	$f_{15}=\frac{{\left(\frac{1}{N}\sum_{n=1}^{N}\sqrt{\left|x_{n}\right|}\right)}^{2}}{\frac{1}{N}\sum_{n=1}^{N}\left|x_{n}\right|}$	Histogram Lower bound	$f_{30}=\max\left(x_{i}\right)-\frac{\left(\max\left(x_{i}\right)-min\left(x_{i}\right)\right)}{2(n-1)}$

## Data Availability

The data that support the findings of this study are available from Korea Hydro & Nuclear Power Central Research Institute but restrictions apply to the availability of these data, which were used under license for the current study, and so are not publicly available. Data are however available from the authors upon reasonable request and with permission of Korea Hydro & Nuclear Power Central Research Institute.
